# Protein Kinase C Is Involved in Vegetative Development, Stress Response and Pathogenicity in *Verticillium dahliae*

**DOI:** 10.3390/ijms241814266

**Published:** 2023-09-19

**Authors:** Dahui Wang, Zhibo Zhao, Youhua Long, Rong Fan

**Affiliations:** College of Agriculture, Guizhou University, Guiyang 550025, China; gzwdh07@126.com (D.W.); zbzhao@gzu.edu.cn (Z.Z.); yhlong3@gzu.edu.cn (Y.L.)

**Keywords:** Potato Verticillium wilt, protein kinase C, pathogenicity, fungicide

## Abstract

Potato Verticillium wilt, caused by *Verticillium dahliae*, is a serious soil-borne vascular disease, which restricts the sustainable development of the potato industry, and the pathogenic mechanism of the fungus is complex. Therefore, it is of great significance to explore the important pathogenic factors of *V. dahliae* to expand the understanding of its pathology. *Protein kinase C* (*PKC*) gene is located in the Ca^2+^ signaling pathway, which is highly conserved in filamentous fungi and involved in the regulation of a variety of biological processes. In the current study, the *PKC* gene in *V. dahliae* (*VdPKC*) was characterized, and its effects on the fungal pathogenicity and tolerance to fungicide stress were further studied. The results showed that the *VdPKC* positively regulated the growth and development, conidial germination, and production of *V. dahliae*, which was necessary for the fungus to achieve pathogenicity. It also affected the formation of melanin and microsclerotia and changed the adaptability of *V. dahliae* to different environmental stresses. In addition, *VdPKC* altered the tolerance of *V. dahliae* to different fungicides, which may be a potential target for polyoxin. Therefore, our results strongly suggest that *VdPKC* gene is necessary for the vegetative growth, stress response, and pathogenicity of *V. dahliae*.

## 1. Introduction

The Ca^2+^ signaling pathway is an important cellular signal transduction cascade that plays a key regulatory role during organismic growth and development [1,2]. The Ca^2+^ signaling pathways of mammals and pathogenic fungi have been systematically studied in recent years. The findings revealed that the proteins related to this pathway mediate the growth and development of pathogenic fungi by rapidly sensing and responding to external stimuli for adapting to different environmental stresses. The proteins also mediate fungal pathogenicity and are relatively conserved among organisms [3,4,5]. The Ca^2+^ signaling pathway of *Saccharomyces cerevisiae* is well studied, and the processes involved in the regulation of Ca^2+^ signaling in this model have been largely elucidated [6,7]. The G proteins of pathogenic fungi are stimulated in response to environmental stresses and, in turn, activate phospholipase C (PLC) in the nucleus, which subsequently hydrolyzes phosphatidylinositol 4,5-bisphosphate (PIP2) to generate inositol 1,4,5-trisphosphate (IP3) and diacylglycerol (DAG). IP3 mobilizes the intracellular Ca^2+^ stores to release Ca^2+^, which acts synergistically with DAG to activate protein kinase C (PKC) for subsequent activation of the downstream signaling cascades [3,8,9]. Therefore, as the activation site of a series of signaling cascades, PKC plays an important role in Ca^2+^ signaling [2,10,11,12].

Conventional PKCs have cysteine-rich C1 and C2 domains at the C-terminus and an HR1 domain at the N-terminus. The regulatory region of the C1 domain can bind DAG/phorbol esters, and the C2 domain mediates Ca^2+^-dependent binding to the membrane lipids, phosphatidylserine (PS), and PIP2. The C-terminus contains a serine/threonine kinase domain and a hydrophobic tail with an Asn-Phe-Asp (NFD) motif. The phenylalanine in the NFD motif cooperates with the ATP substrate to activate PKC [13,14,15,16]. The homologs of *PKC* have been studied using both *PKC* knockout techniques and PKC inhibitors. Previous studies on phytopathogenic fungi have revealed that conidial germination and the formation of appressoria in *Colletotrichum trifolii* are inhibited by PKC-specific inhibitors. It has been reported that the formation of appressoria is disrupted in *PKC* knockout mutants. They are incapable of invading hosts with intact epidermal tissue and exhibit reduced virulence in alfalfa [17]. *PKC* is a regulator of the light-responsive gene, *WC-1* (blue light photoreceptor white collar 1), in the filamentous fungus *Neurospora crassa* [18]. The *PKC* gene of *Aspergillus nidulans* is involved in the pathway related to the preservation of cell wall integrity and mediates the production of penicillin, polarized growth, morphogenesis, formation of septa, and apoptosis [19,20]. Previous studies have additionally demonstrated that *PKC* is a promising antifungal drug target [12]. However, the effects of *PKC* on the pathogenicity, conidial formation, and responses of *Verticillium dahliae* to different environmental stresses remain to be investigated to date.

Potato Verticillium wilt, also known as potato early death disease or precocious ripening disease, is caused by *V*. *dahliae*, and is one of the most economically destructive diseases that affect the production of potato [21,22]. During the initial stages of Potato Verticillium wilt, the lower leaves begin to yellow and expand towards the apex, and the edges of the diseased leaves turn brown and dry. Examination of the longitudinal sections of the stem reveals the browning and necrosis of the vascular bundle system, which eventually leads to the wilting of the entire plant, abnormal development, and early death [23,24,25]. The wide range of hosts and the production of melanin pigment by pathogenic *V. dahliae* contribute to its defense against environmental stresses and high drug resistance, which, together with various other concerns, such as crop commissures, has led to the suboptimal treatment of Verticillium wilt from the point of view of controlling *V*. *dahliae* [26,27,28]. The identification and functional elucidation of the genes involved in the pathogenesis of *V*. *dahliae* is an important aspect of research studies aimed at the prevention and treatment of Potato Verticillium wilt. For instance, previous studies have demonstrated that genes related to signal transduction (*VdCSIN1* [29]), adaptation to environmental stress (*VdPbs2* [30], *VdSsk2* [31], and *VdHog1* [32]), and formation of microsclerotia and melanin synthesis (*Vayg1* [33], *VdCmr1* [34], and *VdSho1* [35]) in *V*. *dahliae* are closely related to fungal pathogenicity. Additionally, subsequent studies have demonstrated that the Ca^2+^ signaling pathway, mitogen-activated protein kinase (MAPK) signaling pathway, and the G protein signaling pathway of *V*. *dahliae* are closely related to the process of pathogenesis [15,36]. The *Crz1* transcription factor is an important downstream regulator of Ca^2+^ signaling in fungi, and the deletion of the *Crz1* ortholog in *V*. *dahliae*, *VdCrz1*, disrupts the formation of microsclerotia and melanin and reduces its virulence in tobacco [37]. The MAPK signaling pathway regulates environmental adaptation, fungal growth and development, and expression of virulence proteins, including the transmembrane mucins that are encoded by the *Msb* gene and are highly conserved in fungal MAPK signaling pathways. Additionally, a previous study reported that the deletion of the *VdMsb* gene of *V*. *dahliae* reduces its virulence in cotton [38]. G protein-mediated signaling pathways regulate cell wall-degrading enzymes and carbon metabolism in pathogens. It has been reported that *V*. *dahliae* mutants lacking the *β*-subunit of G proteins have decreased pathogenicity [39]. These findings imply that the signal transduction pathways of *V*. *dahliae* play a crucial role in the biological processes. Therefore, the identification of disease-related genes in the signal transduction pathways of *V*. *dahliae* and their application for the analysis of possible pesticide targets and resistance genes have immense theoretical and practical significance in the control of *V*. *dahliae*.

PKC is the activation site of the Ca^2+^ signaling cascade, and the identification of the *PKC* gene of *V*. *dahliae* could provide novel insights into its prevention and control. Our previous proteomic analysis and quantitative real time polymerase chain reaction (qRT-PCR) verification results suggested that the *VdPKC* gene of *V*. *dahliae* (*VDAG_09909*) is a promising pathogenic factor during the fungal infection process [40]. In this study, the *PKC* homolog of *V*. *dahliae*, *VdPKC*, was characterized, and further analysis revealed that it was involved in the vegetative growth, formation, and germination of conidia, and played a crucial role in the pathogenicity of *V*. *dahliae*. *VdPKC* also altered the adaptability of *V*. *dahliae* to different environmental stresses, mediated tolerance to different fungicides, and was found to be a potential target of the fungicide polyoxin.

## 2. Results

### 2.1. Sequence Analysis of VdPKC

**Figure 1:**
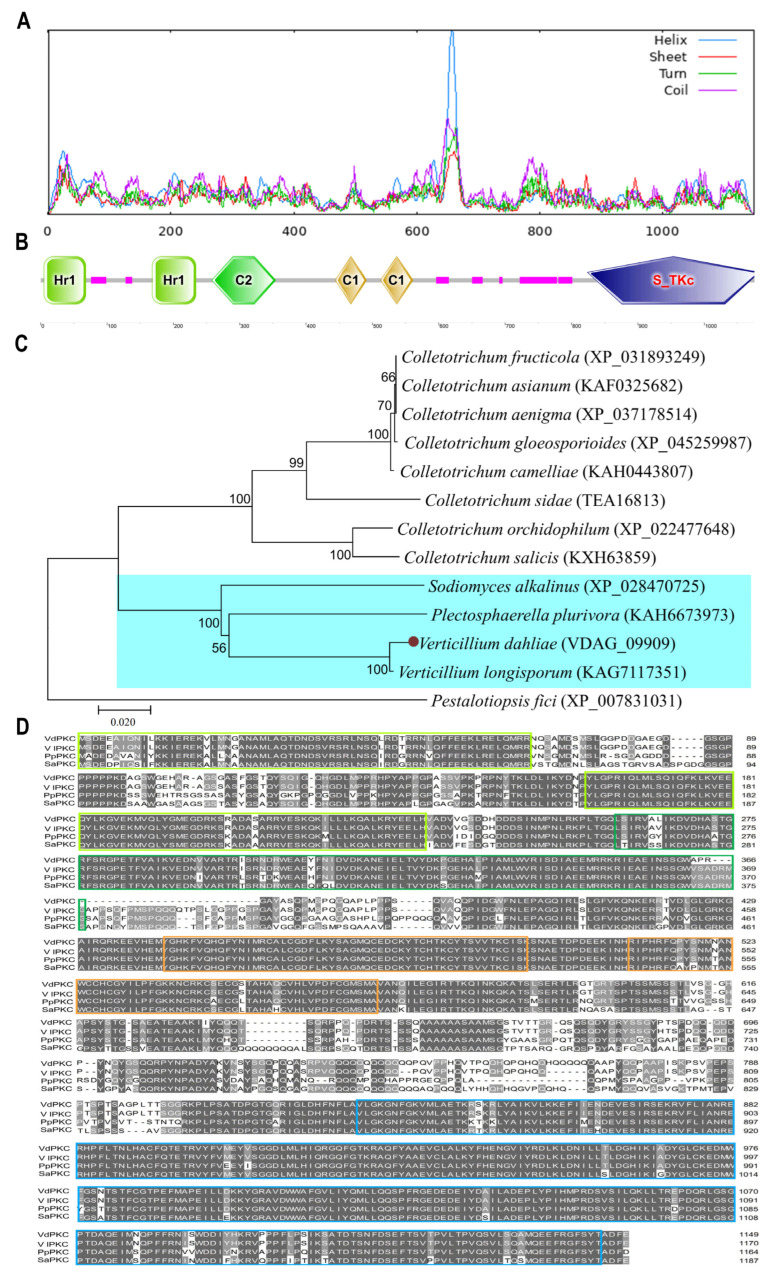
Sequence analysis of the VdPKC protein of *V*. *dahliae*. (**A**) Results of secondary structure prediction. (**B**) Analysis of the domain composition of VdPKC revealed a protein kinase domain. (**C**) Phylogenetic analysis of VdPKC and its homologs. The phylogenetic tree was constructed using the maximum-likelihood method with 1000 bootstraps in MEGA 7.0. The blue box represents a protein that is closely related to VdPKC. (**D**) Multiple sequence alignment of the conserved amino acid sequences of PKC homologs. The residues highlighted in gray and colored boxes indicate conserved amino acids and conserved sites, respectively. The boxes are color-coded according to the domain colors in panel (**B**).

A full-length 3847 bp-long sequence, encoding a protein of 1149 amino acids and a molecular mass of 12.89 kDa, was cloned from the JY strain of *V*. *dahliae* and was designated as *VdPKC*. It showed 100% identity with the sequence in VdLs.17, a type strain of *V*. *dahliae*. The results of protein secondary structure prediction with SOPMA revealed that VdPKC contained four types of secondary structures (Figure 1A), including random coils, α-helices, β-strands, and β-turns, which comprised 50.74% (583 amino acids), 31.77% (365 amino acids), 12.27% (141 amino acids), and 5.22% (60 amino acids), respectively, of the secondary structure content of VdPKC. The results of protein domain prediction with SMART revealed that VdPKC contained HR1 super (HR1), HR1_PKC-like (HR1), C2 superfamily (C2), C1_ScPKC1-like_rpt1 (Pkc1), and C1_ScPKC1-like_rpt2 (Pkc2) domains at the N-terminal, and an STKc_PKC domain at the C-terminal (Figure 1B). The results of phylogenetic analysis with MEGA 7.0 revealed that VdPKC clustered with the PKCs of *V*. *longisporum*, *Plectosphaerella plurivora*, and *Sodiomyces alkalinus*, and were distantly related in the same branch (Figure 1C). A multiple sequence alignment of the PKC orthologs of different fungi was constructed using the Clustal X 2.0 software, and the findings revealed that several key amino acids in the N-terminal HR1, C2, Pkc1, and Pkc2 domains and the C-terminal STKc_PKC domain were highly conserved (Figure 1D).

### 2.2. VdPKC Is Essential for Pathogenicity in Potato

**Figure 2:**
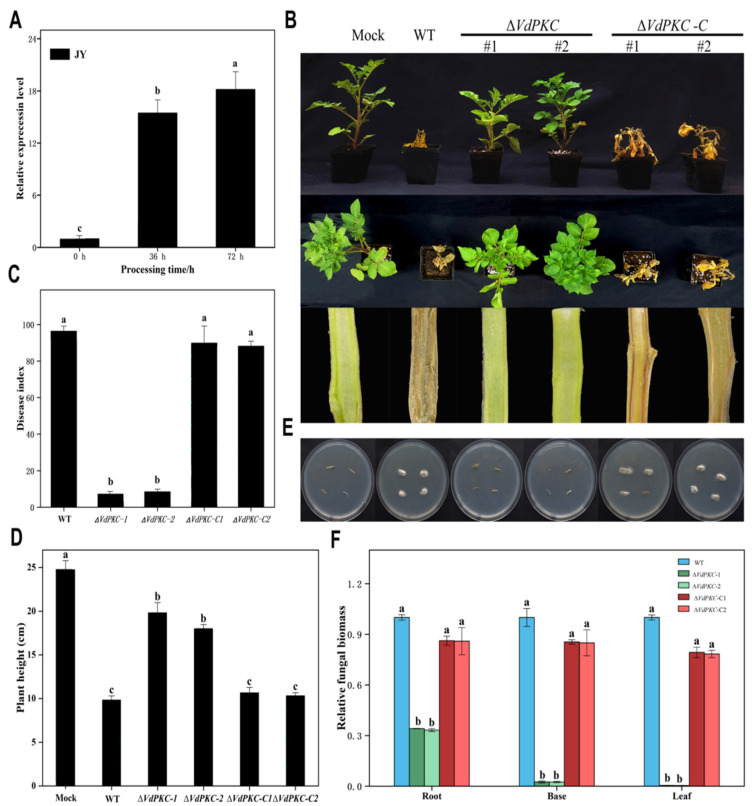
*VdPKC* knockout in *V*. *dahliae* reduces pathogenicity in potato. (**A**) Expression levels of *VdPKC* in potato plants infected with *V*. *dahliae* for 0, 36, and 72 h. The *β-tubulin* gene of *V*. *dahliae* was used as a reference. (**B**) Typical characteristics of Verticillium wilt disease and discoloration of vascular bundles in potato plants inoculated with the spores of knockout Δ*VdPKC* mutants, complementary Δ*VdPKC*-C mutants, and WT (JY) using the root-dipping method for 4 weeks. The potato seedlings in the control setup were inoculated with sterile water. (**C**) Disease indices of potato plants inoculated with the conidia of WT, Δ*VdPKC*, and Δ*VdPKC*-C *V*. *dahliae* for 4 weeks. The grades of disease symptoms have been described in the methods section. (**D**) Heights of potato plants inoculated with the spores of *V. dahliae* mutants and WT strain for 4 weeks. (**E**) Fungal recovery from potato plants inoculated with the conidia of WT, Δ*VdPKC*, and Δ*VdPKC*-C *V*. *dahliae* for 4 weeks. The stem segments were harvested at 4 weeks, plated on potato dextrose agar (PDA) medium, and incubated at 25 °C. The images were captured 3 d post-incubation, and (**F**) qRT-PCR-based measurement of fungal biomass in potato plants inoculated with the conidia of WT, Δ*VdPKC*, and Δ*VdPKC*-C *V*. *dahliae* at 4 weeks. The *actin* gene of potato was selected as a reference. The results were obtained from at least three independent experiments and statistically analyzed using the SPSS statistics software, version 26.0, from IBM. The different letters (a, b, and c) indicate significant differences (*p* < 0.05) determined by one-way analysis of variance (ANOVA) and Tukey’s multiple comparisons test.

In order to determine whether the *VdPKC* gene is involved in the pathogenesis of *V*. *dahliae*, the expression levels of *VdPKC* at the early stage of *V*. *dahliae* infection were determined using the *β-tubulin* of *V*. *dahliae* as reference and conidia at 0 h as control. The results demonstrated that the expression of *VdPKC* was upregulated by 15- and 18-fold at 36 and 72 h, respectively, compared to that at 0 h (Figure 2A), indicating that *VdPKC* may play a role in the pathogenesis of *V*. *dahliae*. In order to confirm this conjecture, *VdPKC* knockout was achieved by homologous recombination, and the Δ*VdPKC* knockout mutants were further investigated (Supplementary Figure S1A,B). The complementary Δ*VdPKC*-C mutants were obtained by transforming a genomic copy of *VdPKC* into the protoplasts of the Δ*VdPKC* mutants (Supplementary Figure S1C). The conidia of the wild-type (WT) *V*. *dahliae* and the Δ*VdPKC* and Δ*VdPKC*-C mutants were collected and inoculated into well-grown six-leaf potato seedlings using the root-dipping method for testing fungal pathogenicity. The results demonstrated that plants inoculated with both the WT and mutant Δ*VdPKC*-C *V*. *dahliae* exhibited obvious symptoms of Verticillum wilt after 4 weeks of inoculation, indicated by the discoloration of vascular bundle tissues. In contrast, plants inoculated with the Δ*VdPKC* mutant exhibited no symptoms of infection and were difficult to distinguish from uninfected plants (Figure 2B). The disease index following infection with Δ*VdPKC* was also significantly lower than that of the WT and mutant Δ*VdPKC*-C (Figure 2C). However, the plant height was affected by Δ*VdPKC* infection (Figure 2D). Correspondingly, *V*. *dahliae* could be successfully re-isolated only from the hypocotyl of plants inoculated with the WT and mutant Δ*VdPKC*-C (Figure 2E). The results of the biomass assay further revealed that the biomass of *V*. *dahliae* decreased in potato plants inoculated with the Δ*VdPKC* mutant (Figure 2F), suggesting that the penetration ability of the Δ*VdPKC* mutant was affected. Therefore, the penetrative ability of the conidia of *V*. *dahliae* through cellulose membranes was subsequently examined, and the results correlated with the aforementioned findings. The results demonstrated that the WT and mutant Δ*VdPKC*-C were capable of penetrating the cellophane for growth, whereas the penetrative ability of the Δ*VdPKC* mutant was impaired, and hyphal penetration was not observed through the cellophane (Supplementary Figure S1D). These results suggested that the *VdPKC* gene is involved in the pathogenesis of *V*. *dahliae*, and the deletion of *VdPKC* affects the penetrative ability of *V*. *dahliae* and reduces its pathogenicity in potato.

### 2.3. VdPKC Mediates the Vegetative Growth of V. dahliae

**Figure 3:**
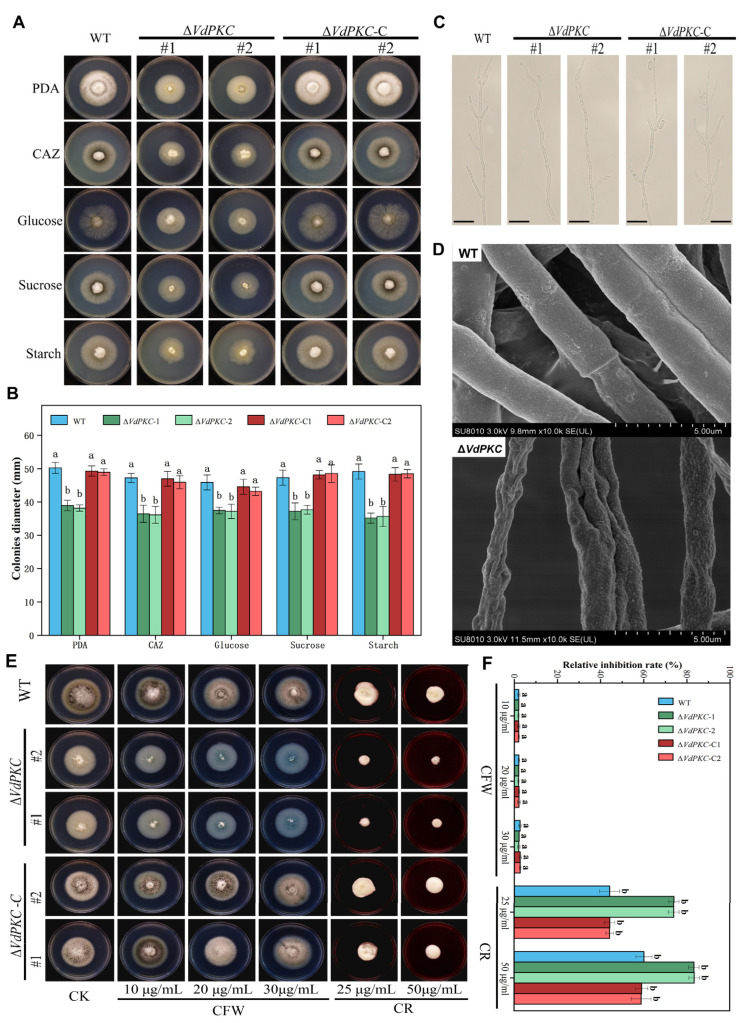
*VdPKC* knockout affected vegetative growth and hyphal formation in *V*. *dahliae*. (**A**) Colony morphology of the WT (JY) *V*. *dahliae*, ∆*VdPKC* knockout mutants, and complementary ∆*VdPKC*-C mutants inoculated in PDA, CAZ, glucose, sucrose, and starch plates for 10 d. (**B**) Colony diameters of the WT, ∆*VdPKC*, and ∆*VdPKC*-C *V*. *dahliae* inoculated in PDA, CAZ, glucose, sucrose, and starch plates for 10 d. (**C**) Mycelial morphology of the WT, ∆*VdPKC*, and ∆*VdPKC*-C *V*. *dahliae*. Scale bar = 20 μm. (**D**) Hyphae of the WT, ∆*VdPKC*, and ∆*VdPKC*-C *V*. *dahliae* by SEM. (**E**) Growth of the WT, ∆*VdPKC*, and ∆*VdPKC*-C colonies cultured for 10 d in PDA containing congo red at concentrations of 25 and 50 μg/mL, or calfluorescent at concentrations of 10, 20, and 30 μg/mL. (**F**) Relative inhibition rates. The results were obtained from at least three independent experiments and statistically analyzed using the SPSS statistics software, version 26.0, from IBM. The different letters (a and b) indicate significant differences (*p* < 0.05) determined by one-way ANOVA and Tukey’s multiple comparisons test.

In order to determine whether the knockout of *VdPKC* affected the growth of *V*. *dahliae*, WT, ∆*VdPKC*, and ∆*VdPKC*-C *V*. *dahliae* were separately inoculated on PDA, Czapek-dox agar (CAZ), glucose, sucrose, and starch plates, and incubated at 25 °C in the dark for studying colony morphology, determination of colony diameter, and microobservation of hyphal morphology. As depicted in Figure 3A, the growth morphology of the ∆*VdPKC* colony was significantly different from that of the WT. The colony diameter of the ∆*VdPKC* mutant was smaller, and the aerial hyphae were significantly reduced compared to those of the WT, and the colony was white in color (Figure 3A). Comparison of the colony diameter further revealed that the colony diameter of the ∆*VdPKC* mutant was significantly reduced. However, the defective phenotype of the ∆*VdPKC* mutant was restored in the ∆*VdPKC*-C mutant, and there was no significant difference between the diameters of the ∆*VdPKC*-C and WT colonies (Figure 3B). Microscopic observation of the hyphal morphology revealed that compared with WT, the ∆*VdPKC* mutant had curved mycelium and fewer threaded branches, ∆*VdPKC*-C returned to WT level (Figure 3C). Subsequent analysis by scanning electron microscopy (SEM) revealed that the mycelial surface of the WT *V*. *dahliae* was complete and smooth, whereas that of the ∆*VdPKC* mutant was wrinkled and had concavities (Figure 3D). The findings implied that the integrity of the cell wall of *V*. *dahliae* was damaged. Therefore, the sensitivity of the WT, ∆*VdPKC*, and ∆*VdPKC*-C *V*. *dahliae* to congo red and calfluorescent was further examined. The results demonstrated that the ∆*VdPKC* mutant was relatively highly sensitive to congo red, and the relative inhibition of ∆*VdPKC* was significantly higher than that of the WT and ∆*VdPKC*-C *V*. *dahlia*. However, the ∆*VdPKC* mutant was insensitive to calfluorescent, and there were no significant differences between the relative inhibition of ∆*VdPKC* and those of the WT and ∆*VdPKC*-C *V*. *dahliae* (Figure 3E,F). These results indicated that the *VdPKC* gene was involved in the establishment of hyphal growth and morphology, affected the integrity of the cell wall, and altered the adaptive capacity of *V*. *dahliae* following exposure to cell wall stimuli.

### 2.4. VdPKC Is Involved in the Formation and Germination of Conidia in V. dahliae

**Figure 4:**
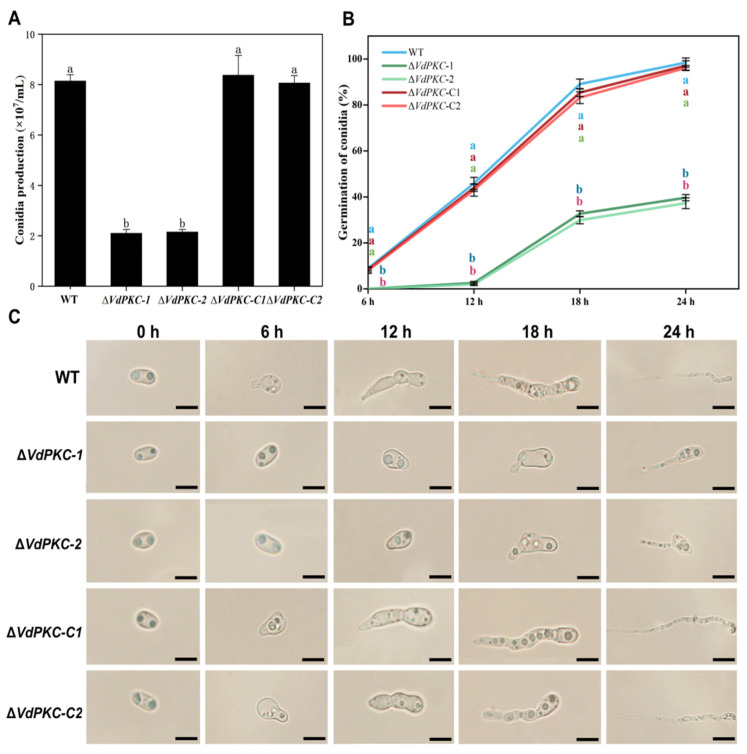
*VdPKC* knockout decreased conidial formation and germination in *V*. *dahliae*. (**A**) Determination of conidial formation in *V. dahliae*. (**B**) Determination of the conidial germination rate of WT and mutant *V. dahliae*, and (**C**) morphological observation of the conidia of *V. dahliae* at 0, 6, 12, 18, and 24 h of germination. Scale bar = 20 μm. The results were obtained from at least three independent experiments and statistically analyzed using the SPSS statistics software, version 26.0, from IBM. The different letters (a and b) indicate significant differences (*p* < 0.05) determined by one-way ANOVA and Tukey’s multiple comparisons test.

In order to elucidate the role of *VdPKC* in the early stages of *V*. *dahliae* infection, the morphological characteristics and formation of conidia were examined, and conidial germination on the hydrophobic surface of coverslips was evaluated over a period of 24 h. The results demonstrated that *VdPKC* had no effect on conidial morphogenesis. However, the formation of conidia was significantly reduced in Δ*VdPKC* mutants compared to those of the WT and Δ*VdPKC*-C *V*. *dahliae* (Figure 4A). The conidia of the WT and Δ*VdPKC*-C mutant began germinating within 6 h, and the rate of germination increased with the extension of the incubation period. Conidial germination was nearly complete at 24 h for the WT and Δ*VdPKC*-C *V*. *dahliae*. The conidia of the Δ*VdPKC* mutants began germinating at 12 h post incubation, and the rate of germination was significantly lower than that of the WT and Δ*VdPKC*-C *V*. *dahliae* at 24 h (Figure 4B,C). Further observation for 48 h revealed that the conidia of the WT and Δ*VdPKC*-C *V*. *dahliae* had fully germinated, whereas the rate of conidial germination of the Δ*VdPKC* mutant was significantly lower than that of the WT and Δ*VdPKC*-C *V*. *dahliae* (Supplementary Figure S2). These results suggested that *VdPKC* is involved in the formation of conidia in *V*. *dahliae* in response to the availability of hydrophobic surfaces for conidial germination.

### 2.5. VdPKC Is Involved in the Formation of Microsclerotia and Melanin in V. dahliae

**Figure 5:**
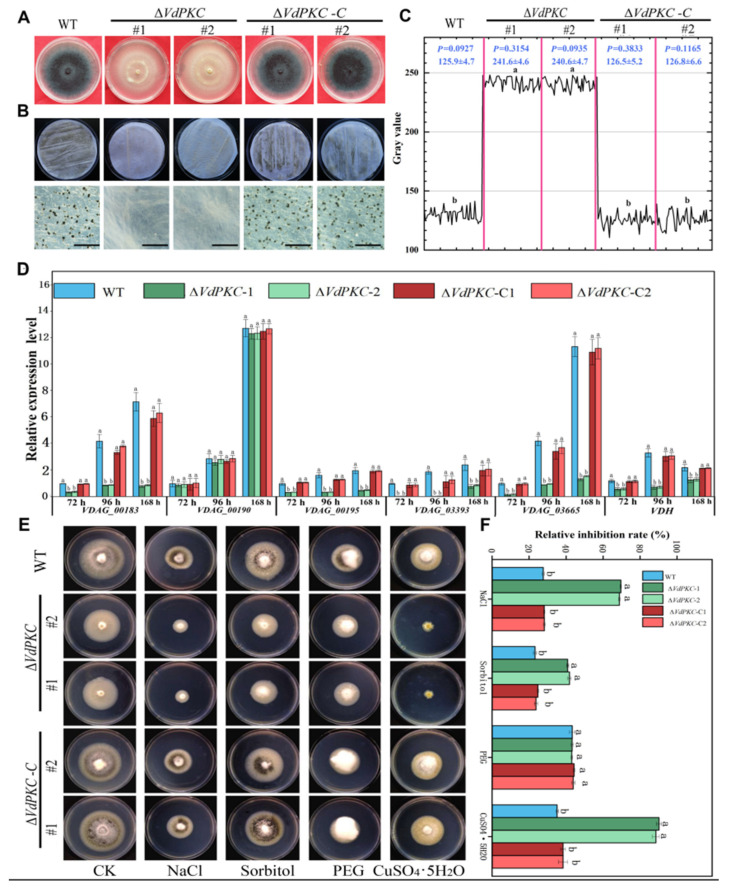
*VdPKC* knockout affected the formation of melanin and microsclerotia in *V*. *dahliae*. (**A**) Colony morphology of WT (JY), ∆*VdPKC* knockout mutants, and complementary ∆*VdPKC*-C mutants inoculated in PDA for 30 d. (**B**) Morphological characteristics and formation of microsclerotia fibers in the conidia of WT, ∆*VdPKC*, and ∆*VdPKC*-C *V*. *dahliae* coated in BMM medium after 14 d of culture. Scale bar = 10 μm. (**C**) Gray value range of (**B**) scanned using the Image J 1.8.0 software. (**D**) qRT-PCR-based detection of the expression levels of genes related to melanin and micronuclei synthesis in WT, ∆*VdPKC,* and ∆*VdPKC*-C *V. dahliae* at 72, 96, and 168 h in BMM medium. The *β-tubulin* gene of *V. dahliae* was selected as reference. (**E**) Morphology of the WT, ∆*VdPKC*, and ∆*VdPKC*-C colonies inoculated in PDA medium containing 0.8 M NaCl, 1.2 M sorbitol, 0.8 M PEG, and 1.2 mM CuSO_4_·5H_2_O, and incubated in the dark at 25 °C for 10 d. (**F**) Relative inhibition rates. Statistical analysis was performed using the SPSS statistics software, version 26.0, from IBM. The different letters (a and b) indicate significant differences (*p* < 0.05) determined by one-way ANOVA and Tukey’s multiple comparisons test.

The results of colony morphology characterization revealed that the ∆*VdPKC* colonies appeared white in color (Figure 3A), which presumably impeded the formation of *V*. *dahliae* microcolonies. The results of qRT-PCR analysis additionally revealed that the expression of *VdPKC* was upregulated during the formation of microcolonies (Supplementary Figure S3). It has been reported that the production of melanin and microsclerotia in *V*. *dahliae* are tightly linked [33]. In order to investigate the effects of *VdPKC* on the formation of microsclerotia and melanin synthesis in *V*. *dahliae*, WT, Δ*VdPKC*, and Δ*VdPKC*-C *V*. *dahliae* were incubated in PDA medium at 25 °C for 30 d. The results demonstrated that the Δ*VdPKC* mutant failed to produce melanin in PDA medium. However, the production of melanin in the WT and Δ*VdPKC*-C *V*. *dahliae* was significantly high (Figure 5A). The conidia of WT, Δ*VdPKC*, and Δ*VdPKC*-C *V*. *dahliae* were collected and cultured on buffered minimal methanol (BMM) medium spread on sterile cellophane for 14 d. The results of microscopic observation revealed that the WT and Δ*VdPKC*-C *V*. *dahliae* formed several melanized microsclerotia. However, the production of microsclerotia or melanin was not observed in the Δ*VdPKC* mutant (Figure 5B). Analysis of the gray-scale curves using Image J 1.8.0 software verified that the synthesis of microsclerotia in the Δ*VdPKC* mutant was defective (Figure 5C). Using the *β-tubulin* gene of *V*. *dahliae* as a reference, the expression levels of genes involved in microsclerotia formation and melanin synthesis in *V*. *dahliae* were determined by qRT-PCR analysis. The findings revealed that the expression levels of *VdPks1*, *VdPks1*, *VdBrn2*, *VdCmr1*, *VdScd1*, *VdBrn1*, and *VDH1* were downregulated to varying degrees in the Δ*VdPKC* mutant. However, the expression levels of these genes in the Δ*VdPKC*-C mutant were restored to those of the WT (Figure 5D). The results of the environmental stress assays revealed that the ∆*VdPKC* mutant was highly sensitive to NaCl, sorbitol, and CuSO_4_·5H_2_O (Figure 5E), and the relative inhibition rates were significantly higher than those of WT and Δ*VdPKC*-C *V*. *dahliae* (Figure 5F). These results indicated that *VdPKC* affected the formation of microsclerotia and melanin in *V*. *dahliae* by regulating the transcription of genes involved in these processes, and the deletion of *VdPKC* altered the resilience of *V*. *dahliae* to different environmental stresses.

### 2.6. VdPKC Is Involved in the Resistance of V. dahliae to Different Fungicides

**Figure 6:**
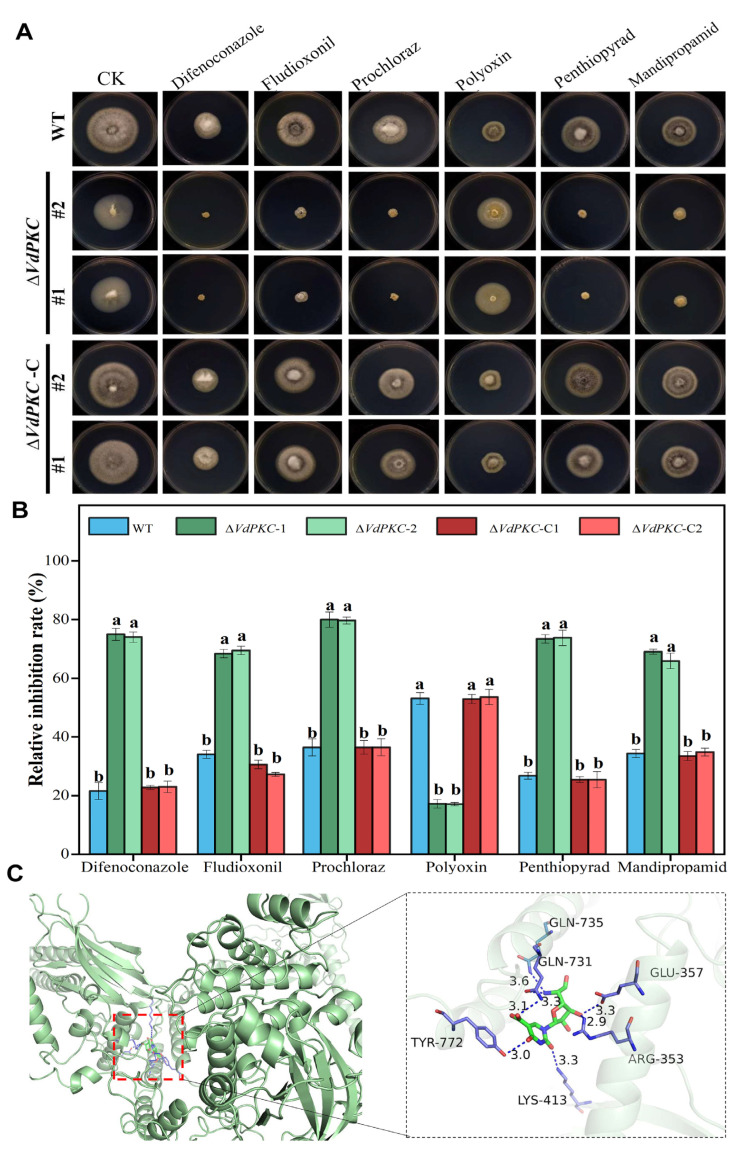
*VdPKC* was involved in the tolerance of *V*. *dahliae* to different fungicides. (**A**) Colony morphology of the WT (JY), ∆*VdPKC* knockout mutant, and ∆*VdPKC*-C complementary mutant cultured in PDA medium containing 5 μg/mL difenoconazole, 5 μg/mL fludioxonil, 0.4 μg/mL prochloraz, 10 μg/mL polyoxin, 8 μg/mL penthiopyrad, or 0.6 μg/mL mandipropamid for 10 d. (**B**) Relative inhibition rates. (**C**) Three-dimensional representation of the binding pose of polyoxin in the active site of VdPKC. The data were statistically analyzed using the SPSS statistics software, version 26.0, from IBM. The different letters (a and b) indicate significant differences (*p* < 0.05) determined by one-way ANOVA and Tukey’s multiple comparisons test.

The aforementioned results demonstrated that *VdPKC* improved the adaptation of *V*. *dahliae* to different environmental stresses. At present, fungicides are one of the most direct and effective methods for controlling *V*. *dahliae* infections [41,42]. Therefore, the tolerance conferred by *VdPKC* to different fungicides was analyzed in this study. The results demonstrated that the ∆*VdPKC* mutant exhibited a high susceptibility to fungicides, and the relative inhibition rates in PDA medium containing difenoconazole, fludioxonil, prochloraz, penthiopyrad, and mandipropamid increased significantly. However, the ∆*VdPKC* mutant exhibited a high tolerance to polyoxin, and the relative inhibition rate was significantly reduced. The tolerance of the ∆*VdPKC*-C mutant was restored to that of the WT (Figure 6A,B). These findings revealed that *VdPKC* was regulated the tolerance of *V*. *dahliae* to different fungicides, and *VdPKC* knockout increased the tolerance of *V*. *dahliae* to polyoxin. The interactions between the fungicides and the VdPKC protein were subsequently determined by molecular docking. To this end, molecular models of the fungicides were searched and retrieved from the PubChem database (Supplementary Figure S4), and a model of the VdPKC protein was constructed using ROBETTA (Supplementary Figure S5). The results demonstrated that polyoxin could spontaneously form complexes with the active site of VdPKC (Supplementary Figure S6) and had a superior free energy binding of −5.6 kcal/mol (Supplementary Table S2). Further analysis revealed that polyoxin interacted with Tyr772, Gln735, Gln731, Glu357, Arg353, and Lys413 of VdPKC, and the hydrogen bond distances were 3.0, 3.3, 3.6, 3.3, 2.9 and 3.3 Å, respectively (Figure 6C). These findings indicated that VdPKC participates in the response of *V*. *dahliae* to different fungicides and can be potentially targeted by polyoxin.

## 3. Discussion

PKCs are involved in the Ca^2+^ signaling pathway and play important roles in fungal growth, development, and pathogenicity, and are potential targets of antifungal drugs [11,12,14,43]. PKCs comprise an important class of pathogenic factors, and studies have confirmed that they are involved in the pathogenesis of a variety of phytopathogenic fungi [16,20]. STKc_PKC is the catalytic domain of PKCs, and HR1, C2, Pkc1, and Pkc2 are the regulatory domains [44]. Previous studies have reported that the two orthologs of PKC in yeast, Pkc1 and Pkc2, operate in a redundant manner to control essential functions, including morphogenesis and cell wall biosynthesis [45,46]. The two HR1 repeats at the N-terminus bind to the active GTPases, Rho1 and Rho2, and increase the stability of Pkc1 and Pkc2 [11,47,48,49]. The *PKC* gene of *V*. *dahliae* was studied in this study, and sequence analysis revealed that the complete sequence of *VdPKC* was 3847 bp long and encoded a protein of 1149 residues. The VdPKC protein contained four types of secondary structures, namely, random coils, α-helices, β-strands, and β-turns. The findings revealed that VdPKC was closely related to the PKC proteins of *V*. *longisporum*, *P*. *plurivora*, and *Sod*. *alkalinus*, and contained the HR1, C2, Pkc1, Pkc2, and STKc_PKC domains. Additionally, the key amino acid sequences in each domain were highly conserved across the different fungal genera, suggesting that the functions of VdPKC could be similar to those of the homologous proteins. The findings provide novel insights for future studies on *VdPKC*.

qRT-PCR analysis was subsequently performed to determine whether the expression of *VdPKC* was induced during infection. The findings revealed that the expression levels of *VdPKC* increased continually during the process of infection with *V*. *dahliae*, suggesting that *VdPKC* was involved in the pathogenesis of *V*. *dahliae*. Therefore, the effect of *VdPKC* on the pathogenicity of *V*. *dahliae* was analyzed by constructing knockout and complementary mutants of *VdPKC*. As expected, the findings revealed that the disease index of Verticillium wilt reduced significantly in potato plants inoculated with ∆*VdPKC*. Morphological observation of the vascular bundle tissues of longitudinally dissected potato stems revealed that the vascular bundles were less discolored, and the color was more similar to the white color of the empty control inoculated with clean water. However, the vascular bundle tissues of potato plants inoculated with WT and ∆*VdPKC*-C *V*. *dahliae* exhibited significant browning, which could be related to the reduced rate of conidial germination and defective penetration capacity of *V*. *dahliae*. Previous studies have demonstrated that *V*. *dahliae* invades host plants via the root system. The microsclerotia or conidia of *V*. *dahliae* germinate to form infectious mycelia that contact the host epidermis and subsequently invade and infect host tissues [50,51]. Previous studies have demonstrated that *V*. *dahliae* mutants with knockout of the *VdSho1* gene that encodes a transmembrane protein and the *VdCSIN1* regulatory factor were incapable of penetrating cellophane, and their pathogenicity was reduced in cotton. Further investigation revealed that the *VdCSIN1* gene is induced and expressed during the invasion of *V*. *dahliae* [29,35]. The *VdGAL4* gene of *V*. *dahliae* encodes a glycosidase hydrolase and has been shown to cause a reduction in the rate of conidial germination, loss of penetrative ability on cellophane, and reduction of pathogenicity in cotton [52]. Detection of the biomass of *V. dahliae* in the roots, stems, and leaf tissues of potato also revealed that the biomass of *V. dahliae* was significantly reduced in potato tissues inoculated with the ∆*VdPKC* mutant, whereas the biomass of the ∆*VdPKC*-C mutant was restored to that of the WT *V. dahliae*. This indicated that the deletion of the *VdPKC* gene decreased the rate of conidial germination and impaired the penetrative ability of *V*. *dahliae*, which reduced the pathogenicity of *V. dahliae* in potato. Previous studies have demonstrated that the growth and development of *V. dahliae* also contribute to its pathogenicity [53,54,55]. Analysis of the growth and development of *V*. *dahliae* revealed that the diameter of the ∆*VdPKC* colony was reduced in PDA and CAZ media, and its growth was significantly retarded in a medium containing different carbon sources. This indicated that the effect of the *VdPKC* gene on the growth and development *V. dahliae* also contributed to its reduced pathogenicity in potato.

Microscopic observation of the hyphal morphology revealed a reduction in hyphal whorl branching in the ∆*VdPKC* mutant, and the hyphal surface appeared uneven and tortuous, indicating that the knockout of *VdPKC* affected hyphal formation in *V*. *dahliae*. It has been reported that the conidia of *V*. *dahliae* germinate at the tips of the verticillium branches and then spindle by apical budding [56]. Therefore, the reduction of branching in *V*. *dahliae* via the knockout of *VdPKC* is the primary reason underlying the reduction in conidial formation. Congo red and calfluorescent are commonly used cell wall stress factors, of which congo red inhibits microfibril arrangement by binding to *β*-1,3-glucans in the cell wall, and calfluorescein inhibits the assembly of *β*-1,4-glucan and chitin in cell walls by binding to chitin [57]. It has been reported that the loss of function of *Pkc1P* in yeast leads to the destruction of cell walls, which results in cellular rupture [58]. In this study, the results of SEM analysis revealed that the hyphal surface of the ∆*VdPKC* mutant was wrinkled and folded inwards, while that of the WT fungi appeared smooth and complete. This led to the speculation that the integrity of the cell wall of *V*. *dahliae* was damaged following *VdPKC* knockout. The results demonstrated that although ∆*VdPKC* was insensitive to calfluorescent, its sensitivity to congo red was significantly high, which indicated that *VdPKC* affected the integrity of the cell wall in *V*. *dahliae*.

Previous studies have demonstrated that filamentous fungi have very complex environmental stress response systems, which can respond rapidly by sensing the changes in the external environment, including salt stress, osmotic stress, drought stress, and heavy metal contamination. These response pathways primarily include the Ca^2+^ signaling, G protein, and MAPK signaling pathways, among others [15,45]. Of these, the Ca^2+^ signaling pathway is essential for fungal defense against diverse stresses [13]. *V*. *dahliae* is the dominant pathogen responsible for verticillium wilt and has a strong environmental adaptability that could be attributed to its superior defense mechanism, which can defend and protect against diverse environmental stresses [59,60]. The sensitivity of *V*. *dahliae* to various environmental stresses was examined in this study, and the results demonstrated that *VdPKC* affected the ability of *V*. *dahliae* to adapt to different environmental stresses under natural growth conditions. The findings also revealed that the knockout of *VdPKC* inhibited mycelial growth and significantly increased the sensitivity of *V*. *dahliae* to salt, osmotic, and heavy metal stresses, thereby reducing its ability to survive under natural conditions. *VdPKC* did not affect the sensitivity of *V*. *dahliae* to drought stress, which could be attributed to the presence of potential stress response-related pathways in *V*. *dahliae* that are involved in drought stress response. These findings indicated that *VdPKC* was not involved in the drought stress response pathway, which further illustrates the complexity of the environmental stress response system that confers a superior defense ability under diverse environmental conditions. Notably, previous studies have demonstrated that *V. dahliae* primarily thrives as stress-resistant microsclerotia in adverse environments. The formation of microsclerotia is accompanied by the accumulation of melanin, which finally transforms into mature black microsclerotia with strong stress resistance [30,33,43,61]. The present study revealed that *VdPKC* regulates the transcription of melanin and sclerotia-related genes (Figure 5D), thereby affecting the formation of microsclerotia and melanin. Further studies are necessary to determine whether *VdPKC* is directly involved in the tolerance of *V*. *dahliae* to adverse conditions or is indirectly involved via microsclerotia.

Using different fungicides is currently one of the most direct and effective methods for the prevention and control of *V*. *dahliae* infections [41]. Ca^2+^ is a ubiquitous intracellular secondary messenger, and the Ca^2+^ signaling pathway is highly conserved among organisms, playing an important role in regulating drug resistance [2,13]. *Crz1* is essential for responding to Ca^2+^ signaling, and the loss of the *Crz1* gene in yeast increases their sensitivity to fluconazole [62]. The *PKC* gene partakes in the Ca^2+^ signaling pathway and plays a regulatory role in fungal drug resistance. Therefore, the present study aimed to determine the inhibitory effect of fungicides on WT and mutant *V*. *dahliae*. The results demonstrated that the *VdPKC* gene enhanced the sensitivity of *V*. *dahliae* to difenoconazole, fludioxonil, prochloraz, penthiopyrad, and mandipropamid and enhanced the tolerance of *V*. *dahliae* to polyoxin. The binding modes of the fungicides in the active site of VdPKC were determined by molecular docking. The findings revealed that polyoxin formed a stable complex with the active site of VdPKC with superior free energy of binding, which indicated that *VdPKC* could be potentially targeted by polyoxin. Polyoxin is a metabolite produced by *Streptomyces aureus*, which mainly interferes with the synthesis of fungal cell wall to affect the growth of fungi [63]. The results of this study show that *VdPKC* destroys the integrity of the cell wall of *V. dahliae* (Figure 3D–F). Therefore, it is speculated that this fungicide acts on *V. dahliae* by combining with *VdPKC* to destroy the cell wall of the strain, thereby affecting the growth of the strain, and achieving the purpose of prevention and control. However, as the fungicides are incapable of targeting *VdPKC* following knockout, mycelial growth was not affected by fungicide treatment. This finding serves as a basis for the comprehensive investigation of the mechanism of *V*. *dahliae* infections and the subsequent development of novel fungicides. Additionally, the findings provide a theoretical basis for the development of novel strategies for controlling *V*. *dahliae*.

In this study, bioinformatics analysis was used to deepen the understanding of the obtained *VdPKC* gene, and knockout and complementary mutants were constructed to elucidate the involvement of *VdPKC* in the growth, development, and pathogenesis of *V*. *dahliae*. The findings revealed that *VdPKC* altered the tolerance of *V*. *dahliae* to different environmental stresses and fungicides and could be a potential target of the fungicide polyoxin. This study lays a foundation for further exploring the pathogenic mechanism of *V*. *dahliae* and provides a new theoretical basis for the subsequent prevention and control of *V*. *dahliae* and provides more reference for the subsequent research and development of new fungicides.

## 4. Materials and Methods

### 4.1. Plants, Microbial Strains, and Culture Conditions

The JY strain of WT *V*. *dahliae* was cultured in PDA medium (200 g/L potato, 20 g/L glucose, and 15 g/L agar) at 25 °C [33]. The pOSCAR and pA-Hyg-OSCAR knockout plasmids and the pFL2 complementary vector were donated by Professor Xiaoping Hu, School of Plant Protection, Northwest A & F University, China. The susceptible “Favorita” variety of potato (*Solanum tuberosum* L) was used for the experiments. The potato seedlings were planted in sterilized soil at a temperature of 28/25 °C (day/night) and a day/night photoperiod of 8 h/16 h [8].

### 4.2. Gene Cloning and Sequence Analysis

The sequence of the *VdPKC* gene (accession number: *VDAG_09909*) was retrieved from the genome of *V*. *dahliae* VdLs.17 in the JGI (https://genome.jgi.doe.gov/Verda1/Verda1.home.html (accessed on 11 March 2023)) database, and the *VdPKC*-F/R primers were designed by primer software, version 6.0. The full-length sequence of *VdPKC* was amplified by PCR from the genomic DNA of the JY strain. The secondary structure of VdPKC was analyzed using the SOPMA tool (https://npsa-pbil.ibcp.fr/cgi-bin/secpred_sopma.pl (accessed on 18 March 2023)). The degree of residue conservation in VdPKC was analyzed by amino acid sequence alignment using the Clustal X 2.0 software. Phylogenetic analysis of VdPKC and other homologous PKC proteins was performed using the neighbor-neighbor method in MEGA software, version 7.0.

### 4.3. Gene Knockout and Construction of Complementary Mutants

The knockout mutants were constructed by *Agrobacterium tumefaciens*-mediated transformation [32,33,64]. Using the genomic DNA of *V. dahliae* JY strain as the template, the upstream and downstream gene sequences of *VdPKC* gene were searched and downloaded as homologous arm fragments to construct the knockout vector. The upstream and downstream homologous arms of *VdPKC* were amplified by using *VdPKC*-up-F/R and *VdPKC*-down-F/R primers. Using the pA-Hyg-OSCAR plasmid as the template, the hy gromycin gene was amplified with *hph*-F/R primers. The knockout vector was constructed as described above, and the constructed knock-out vector was transformed into *Agrobacterium*-mediated competent cells. The deletion mutant was constructed through agrobacterium tumefaciens-mediated genetic transformation, and the knockout mutant was obtained through resistance screening and PCR verification. Using *V. dahliae* genomic DNA as a template, the *VdPKC* complementary fragment containing the promoter sequence was amplified by using *VdPKC*-C-F/R primers and connected with the enzyme tangent vector pFL2 to obtain the complementary vector. The complementary vector was introduced into the protoplast of the knockout mutant through polyethylene glycol (PEG)-mediated genetic transformation and the complementary mutant was obtained through resistance screening and PCR verification [33]. The primers used in this study are listed in Supplementary Table S1.

### 4.4. Pathogenicity and Penetration Assays

In order to evaluate the role of the *VdPKC* gene in the pathogenicity of *V*. *dahliae*, the roots of six-leaf potato seedlings were inoculated with a conidial suspension of the JY strain of *V*. *dahliae* and incubated by shaking at 100 rpm at 25 °C. The RNA was extracted after 0, 36, and 72 h of incubation, and the cDNA was generated by reverse transcription. The expression of the *VdPKC* gene was detected by qRT-PCR using the *β-tubulin* gene of *V*. *dahliae* as a reference [33]. The relative gene expression levels were calculated using the 2^−ΔΔCt^ method [65]. The conidia of the WT, ∆*VdPKC*, and ∆*VdPKC*-C *V*. *dahliae* were collected in sterile water, and the concentration was adjusted to 10^7^ conidia/mL. Healthy six-leaf potato seedlings of similar size and growth status were selected as inoculation subjects. The root dipping method was used for inoculation [37], and the healthy potato seedlings were removed from the seedling pots. Their roots were immersed in the conidial suspension for 10 min, following which they were removed and replanted in sterile soil. The seedlings soaked in sterile water for the same time were used as the blank control. The onset of verticillium wilt was observed, and the disease index was statistically evaluated after 4 weeks of inoculation [31,40]. The tissues at the base of the potato stems were collected after 4 weeks of inoculation. The *actin* gene of potato was used as the internal reference gene, and the potato plants treated with sterile water were used as the blank control. The biomass of *V*. *dahliae* was estimated by qRT-PCR using specific *Vd*-F/R detection primers for *V*. *dahliae* [33]. A 30 μL aliquot of the collected conidia was separated and dropped onto the center of PDA plates coated with a cellulose membrane. The growth performance was recorded after 3 d of incubation at 25 °C by capturing the images, and the cellulose membrane was removed. The cultures were continued under seal at 25 °C for 5 d, and the images were captured for recording the phenotype of *V*. *dahliae* [31]. All the experiments were performed in triplicate. The primers used are enlisted in Supplementary Table S1.

### 4.5. Vegetative Growth Assay

In order to evaluate the effect of the *VdPKC* gene on the vegetative growth of *V*. *dahliae*, WT, ∆*VdPKC*, and ∆*VdPKC*-C *V*. *dahliae* were inoculated on PDA, CAZ, and media containing sucrose, glucose, and starch, and cultured at 25 °C in the dark. The diameters of the colonies were measured at 3, 6, 9, and 12 d. Multiple sterile glass slides were inserted obversely around the growing mycelia, and the morphological characteristics of the mycelia were observed by microscopy and SEM. All the experiments were performed in triplicate.

### 4.6. Conidial Yield and Determination of Germination Rate

For the conidial yield assay, the WT, ∆*VdPKC*, and ∆*VdPKC*-C colonies cultured in PDA medium for 7 d were activated by rinsing with sterile water. The conidia were collected and inoculated in 50 mL potato dextrose broth (PDB) liquid medium containing a final concentration of 10^4^ conidia/mL and incubated by shaking at 150 rpm for 5 d at 25 °C. The number of spores was microscopically recorded using a hemocyte-counting plate, and the mean values and standard deviations were calculated. A 10 μL aliquot of the conidial suspension was collected at the same time and placed on a sterile slide, covered with a cover slip, and incubated at 25 °C. Conidial germination was observed under a microscope after 0, 6, 12, 18, 24, and 48 h of incubation, and the mean values and standard deviations were calculated. All the assays were performed in triplicate.

### 4.7. Observation of Microsclerotia Formation

In order to analyze the effect of the *VdPKC* gene on the formation of microsclerotia in *V*. *dahliae*, the WT conidia were collected and 30 μL of the sample was sieved and coated on BMM plates (5 g glucose, 0.2 g NaNO_3_, 0.52 g KCl, 0.52 g MgSO_4_·7H_2_O, 1.52 g KH_2_PO_4_, and 18 g agar per liter) coated with a sterile cellulose membrane. Samples were obtained after 72, 96, and 168 h of incubation at 20 °C in the dark. The sample obtained after 72 h of incubation was selected as the control, and *β-tubulin* was used as the reference gene. The expression of *VdPKC* was subsequently detected. The conidial suspensions of WT, ∆*VdPKC*, and ∆*VdPKC*-C *V*. *dahliae* were collected, and the concentrations were adjusted to 10^7^ conidia/mL, following which 30 μL of the conidial suspensions were separately coated on BMM plates coated with a sterile cellulose membrane and incubated at 20 °C in the dark for 14 d [31]. The number of microsclerotia was counted and photographed. The images were processed using the Image J 1.8.0 software to calculate the gray values and significance of the microsclerotia [65]. Samples were also collected after 72, 96, and 168 h of incubation to compare the expression levels of genes related to microsclerotia formation (*VDH1* [66]) and melanin synthesis (*VdPks1* (*VDAG_00190*), *VdBrn2* (*VDAG_00183*), *VdCmr1* (*VDAG_00195*), *VdScd1* (*VDAG_03393*), and *VdBrn1* (*VDAG_03665*) [31,33,35] in WT, ∆*VdPKC*, and ∆*VdPKC*-C *V*. *dahliae*. The primers used in the experiments are enlisted in Supplementary Table S1.

### 4.8. Analysis of Abiotic Stress Response

In order to analyze the resistance conferred by the *VdPKC* gene to various abiotic stresses, WT, ∆*VdPKC*, and ∆*VdPKC*-C *V*. *dahliae* were inoculated in PDA plates supplemented with different abiotic stress reagents, including cell wall stress reagents (25 and 50 μg/mL congo red; 10, 20, and 30 μg/mL calfluorescent), environmental stress reagents (1.2 M sorbitol, 0.8 M PEG, 0.8 M NaCl, and 2 mM CuSO_4_·5H_2_O), and chemical fungicides (5 μg/mL difenoconazole, 5 μg/mL fludioxonil, 0.4 μg/mL prochloraz, 10 μg/mL polyoxin, 8 μg/mL penthiopyrad, and 0.6 μg/mL mandipropamid). *V*. *dahliae* was subsequently cultured at 25 °C in the dark for 10 d, and the growth and diameters of the colonies were determined. The ratio of the colony diameter at a given concentration of a stress reagent to that in the absence of the reagent was calculated. All the experiments were performed in triplicate.

### 4.9. Molecular Docking

The three-dimensional structure of the VdPKC protein was constructed using the ROBETTA server. The reliability of the modeled structure was validated using the ERRAT program in SAVES (http://servicesn.mbi.ucla.edu/ (accessed on 24 March 2023)) and by Ramachandran plot analysis. The fungicides were docked to the VdPKC protein using AutoDock Vina version 1.1.2. The structures of the ligands were searched from the PubChem (http://pubchem.ncbi.nlm.nih.gov (accessed on 25 May 2023)) database and retrieved in sdf format. The structures were imported to the MM2 module of Chemdraw 3D for energy minimization, and the lowest energy structures were obtained as mol2 files. The structures were visualized using PyMOL, and MGLTools 1.5.6 was used for removing the water molecules and adding hydrogen atoms. The charges were calculated, and the files were saved in pdbqt format after various processing steps, including the incorporation of non-polar hydrogens. AutoDock Vina version 1.1.2 was used to perform semi-flexible docking of the receptor and ligands. The x, y, and z coordinates of the center of the grid were −14.357, 32.374, and 2.492, respectively, and the size of the grid was 20.0 along the x, y, and z axes. The results of molecular docking were analyzed, and fungicides with superior docking scores were selected. The binding modes of the fungicides in the active site of VdPKC were visualized using PyMOL 1 and Discovery Studio.

### 4.10. Data Analyses

The standard errors were calculated using the SPSS statistical software SPSS (version 16.0). Multiple comparisons among groups were performed by ANOVA with the LSD multiple comparison test in SPSS. The data from the two groups were compared by *t*-tests using SPSS.

## Data Availability

The data of the report result has been uploaded to the iprox database https://www.iprox.cn/page/PSV023.html;?url=1676462571051vGlE accessed on 25 May 2023.

## Supplementary Materials

The following supporting information can be downloaded at: https://www.mdpi.com/article/10.3390/ijms241814266/s1.
