# Supplementary material for: Protein Kinase C Is Involved in Vegetative Development, Stress Response and Pathogenicity in Verticillium dahliae
Source: Int J Mol Sci. 2023 Sep 19;24(18):14266. doi: 10.3390/ijms241814266 (PMC10531995; doi:10.3390/ijms241814266)
Supplement: Supplementary file 1 [file ijms-24-14266-s001.zip › Supplementary Figure S1.pdf]

International Journal of *Molecular Sciences*

MDPI

Article

Protein Kinase C Is Involved in Vegetative Development, Stress Response and Pathogenicity in *Verticillium dahliae*

Dahui Wang, Zhibo Zhao, Youhua Long and Rong Fan *

College of Agriculture, Guizhou University, Guiyang 550025, China; gzwdh07@126.com (D.W.); zbzhao@gzu.edu.cn (Z.Z.); yhlong3@gzu.edu.cn (Y.L.)

* Correspondence: rfan@gzu.edu.cn

Supplementary Figure S1. Generation of *VdPKC* knockout and complementation mutants, and penetration ability assay

To define the specific function of *VdPKC* in *Verticillium dahliae* JY strain, we used the principle of homologous recombination to knockout *VdPKC* in *V. dahliae*, and the knockout vector was constructed as shown in supplementary figure 1A. The successfully constructed knockout vector were transferred into the *V. dahliae* by Agrobacterium mediated genetic transformation [1,2], and 2 normally growing resistant transformants were selected by continuous culture in PDA selection medium containing resistance, which were verified by PCR using adaptor primers up-*Hyg*-F/R and *Hyg*-down-F/R. The results showed that the corresponding gene fragment of interest could be amplified in lanes 1, 2, 5, 6 (Supplementary Figure 1B), and the original *VdPKC* gene was replaced by *Hyg*, indicating successful *VdPKC* knockout, which was named Δ*VdPKC*-1, Δ*VdPKC*-2.

To verify that the defect caused by the Δ*VdPKC* mutants in *V. dahliae* is indeed caused by the knockout of this gene, the *VdPKC* complementation assay was performed in this study by retransferring the *VdPKC* into the Δ*VdPKC* mutants to see whether the complementation of this gene could restore the defective phenotype. The constructed complementation vector was introduced into the protoplasts of the knockout mutants by PEG mediated genetic transformation, and 2 resistant transformants were selected by continuous culture in PDA containing selection medium and verified by PCR using primers *VdPKC*-tg-F/R. The results showed that this primer could amplify the fragment of interest from both the *V. dahliae* and the resistant transformant genomic DNA (Supplementary Figure 1C), indicating successful retro complementation of the *VdPKC*, which was named Δ*VdPKC*-C1, Δ*VdPKC*-C2.

Pathogenicity the results confirmed that the *VdPKC* was involved in regulating *V. dahliae* pathogenicity, and additionally we observed subtle color changes in vascular bundle tissues of potato plants inoculated with the Δ*VdPKC*, which was close to the blank control inoculated with clear water, we speculated that *V. dahliae* penetration ability was affected, so we examined the penetration of *V. dahliae* conidia through cellulose membranes [3]. Consistent with the expected results, inoculation of WT, Δ*VdPKC* and Δ*VdPKC*-C conidia onto PDA medium coated with a sterile cellulose membrane unmasked the cellophane after 3 d and continued incubation for an additional 5 d to observe penetration of the cellophane, showing that WT and Δ*VdPKC*-C were able to penetrate cellophane for growth, whereas *V. dahliae* with knockout of the *VdPKC* were impaired in their ability to penetrate cellophane and no hyphae were observed to penetrate cellophane (Supplementary Figure 1D). Showed that knockout of the *VdPKC* resulted in impaired penetration of *V. dahliae*.

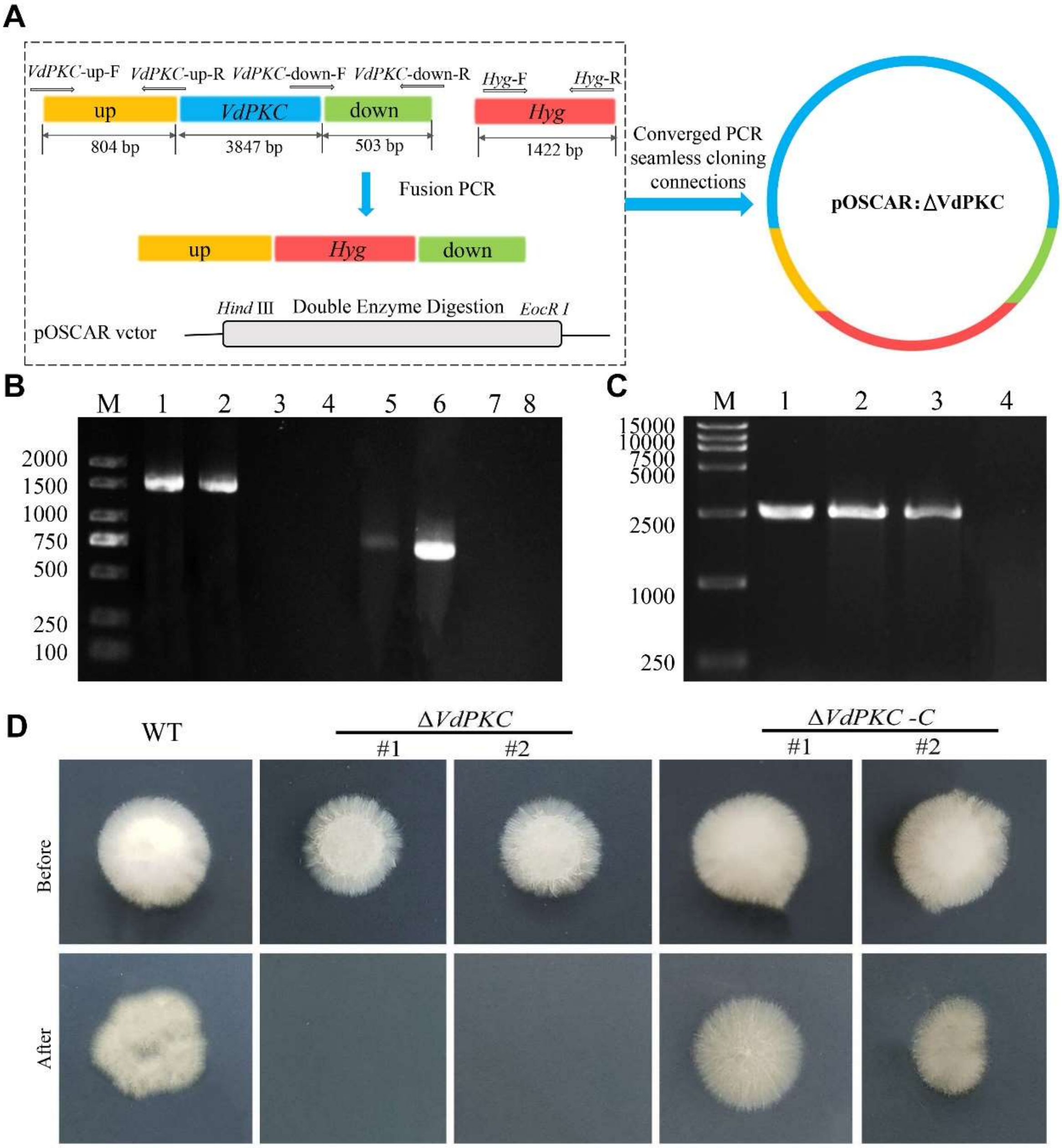

Figure S1. Generation of *VdPKC* knockout and complementation mutants, and penetration ability assay. (**A**) Schematic of *VdPKC* gene knockout in *Verticillium dahliae*. (**B**) Knockout transformants were verified by PCR. M: DL5000 DNA Marker. Lanes 1, 2, 5, 6 are transformant DNA, lanes 3, 7 are *V. dahliae* genomic DNA, lanes 4,8 are negative controls. (**C**) PCR validation of complementation transformants. M: DL15000 DNA Marker. Lanes 1, 2 are transformant genomic DNA, lane 3 is *V. dahliae* genomic DNA, and lane 4 is a negative control. (**D**) penetration ability assay. The first row represented the growth of WT, Δ*VdPKC* and Δ*VdPKC*-C conidia inoculated on PDA medium plated with sterile cellophane for 3 d, and the second row represented the growth of WT, Δ*VdPKC* and Δ*VdPKC*-C on PDA medium for 5 d after unmasking the cellophane

References

1. Wang, Y.L.; Tian, L.Y.; Xiong, D.G.; Klosterman, S.J.; Xiao, S.X.; Tian, C.M. The mitogen-activated protein kinase gene, *VdHog1*, regulates osmotic stress response, microsclerotia formation and virulence in *Verticillium dahliae*. *Fungal Genet. Biol.* **2016**, *88*, 13–23.
2. Fan, R.; Klosterman, S.J.; Wang, C.h.; Subbarao, K.V.; Xu, X.M.; Shang, W.J.; Hu, X.P. *Vayg1* is required for microsclerotium formation and melanin production in *Verticillium dahliae*. *Fungal Genet. Biol.* **2017**, *98*, 1–11.
3. Li, J.J.; Zhou, L.; Yin, C.M.; Zhang, D.D.; Klosterman, S.J.; Wang, B.L.; Song, J.; Wang, D.; Hu, X.P.; Subbarao, K.V.; et al. The *Verticillium dahliae* Sho1-MAPK pathway regulates melanin biosynthesis and is required for cotton infection. *Environ. Microbiol.* **2019**, *21*, 4852–4874.
