# Supplementary material for: Protein Kinase C Is Involved in Vegetative Development, Stress Response and Pathogenicity in Verticillium dahliae
Source: Int J Mol Sci. 2023 Sep 19;24(18):14266. doi: 10.3390/ijms241814266 (PMC10531995; doi:10.3390/ijms241814266)
Supplement: Supplementary file 1 [file ijms-24-14266-s001.zip › Supplementary Figure S2.pdf]

International Journal of
Molecular Sciences

MDPI

Article

Protein Kinase C Is Involved in Vegetative Development, Stress Response and Pathogenicity in *Verticillium dahliae*

Dahui Wang, Zhibo Zhao, Youhua Long and Rong Fan *

College of Agriculture, Guizhou University, Guiyang 550025, China; gzwdh07@126.com (D.W.); zbzhao@gzu.edu.cn (Z.Z.); yhlong3@gzu.edu.cn (Y.L.)
* Correspondence: rfan@gzu.edu.cn

Supplementary Figure S2. Knockout of the *VdPKC* gene affected conidial germination of *Verticillium dahliae*

Continued cultivation of WT, Δ*VdPKC* and Δ*VdPKC*-C conidia until 48 h microscopic observation of the germination revealed that WT and Δ*VdPKC*-C had fully germinated, whereas Δ*VdPKC* conidia still had only a small portion (Supplementary Figure 2). Showed that deletion of vdpkc gene affected the germination of conidia of *V. dahliae*.

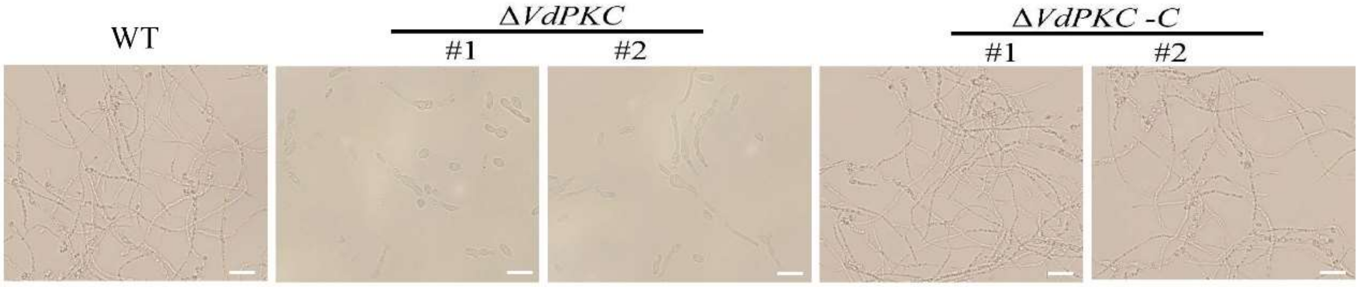

Figure S2. Knockout of the *VdPKC* gene affected conidial germination of *Verticillium dahliae*. The conidia of WT, Δ*VdPKC* and Δ*VdPKC*-C were germinated for 48 h, Bar=50 μm.
