# Supplementary material for: Protein Kinase C Is Involved in Vegetative Development, Stress Response and Pathogenicity in Verticillium dahliae
Source: Int J Mol Sci. 2023 Sep 19;24(18):14266. doi: 10.3390/ijms241814266 (PMC10531995; doi:10.3390/ijms241814266)
Supplement: Supplementary file 1 [file ijms-24-14266-s001.zip › Supplementary Figure S3.pdf]

International Journal of
Molecular Sciences

MDPI

Article

Protein Kinase C Is Involved in Vegetative Development, Stress Response and Pathogenicity in *Verticillium dahliae*

Dahui Wang, Zhibo Zhao, Youhua Long and Rong Fan *

College of Agriculture, Guizhou University, Guiyang 550025, China; gzwdh07@126.com (D.W.); zbzhao@gzu.edu.cn (Z.Z.); yhlong3@gzu.edu.cn (Y.L.)
* Correspondence: rfan@gzu.edu.cn

Supplementary Figure S3. Expression levels of *VdPKC* during microsclerotium formation in *Verticillium dahliae*

To determine whether *VdPKC* gene are involved in regulating the formation of microsclerotia in *V. dahliae*. The *V. dahliae* conidia were collected, spread in BMM medium containing a sterile cellulose membrane, incubated at 20°C in the dark, and sampled at 72, 96, and 168 h, respectively, with 72 h as a control, and *V. dahliae β-tubulin* was used as an reference gene to examine *VdPKC* expression during and microsclerotia formation. The results of qRT-PCR showed that *VdPKC* gene was up-regulated during microsclerotia formation (Supplementary Figure 3). Showed that the *VdPKC* gene is involved in the synthesis of microsclerotia in *V. dahliae*.

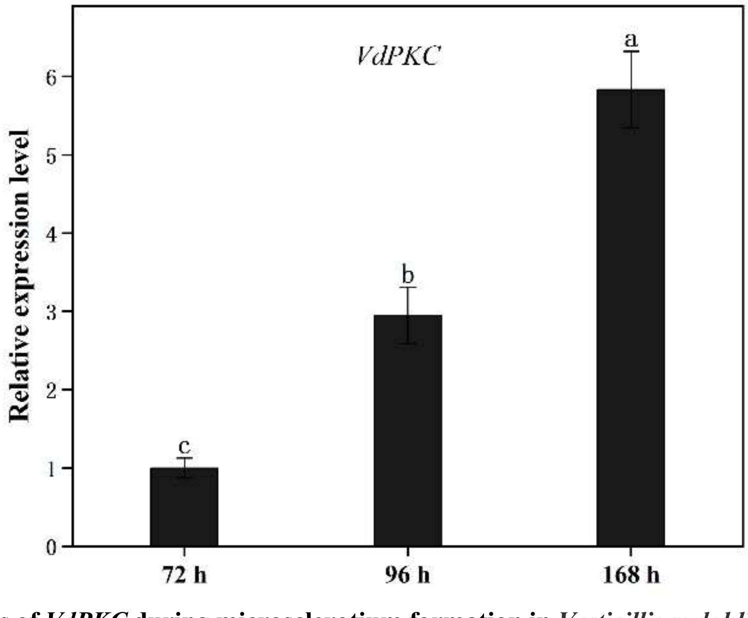

Figure S3. Expression levels of *VdPKC* during microsclerotium formation in *Verticillium dahliae*. Means were calculated from 3 independent replicates, error lines represent standard deviations, and different letters (a and b) indicate significant differences ($P<0.05$).
