# Supplementary material for: Protein Kinase C Is Involved in Vegetative Development, Stress Response and Pathogenicity in Verticillium dahliae
Source: Int J Mol Sci. 2023 Sep 19;24(18):14266. doi: 10.3390/ijms241814266 (PMC10531995; doi:10.3390/ijms241814266)
Supplement: Supplementary file 1 [file ijms-24-14266-s001.zip › Supplementary Figure S5.pdf]

International Journal of *Molecular Sciences*

Article

Protein Kinase C Is Involved in Vegetative Development, Stress Response and Pathogenicity in *Verticillium dahliae*

Dahui Wang, Zhibo Zhao, Youhua Long and Rong Fan *

College of Agriculture, Guizhou University, Guiyang 550025, China; gzwdh07@126.com (D.W.); zbzhao@gzu.edu.cn (Z.Z.); yhlong3@gzu.edu.cn (Y.L.)
* Correspondence: rfan@gzu.edu.cn

Supplementary Figure S5. VdPKC protein model constructed by ROBETTA

The 3D model of the VdPKC protein was generated by modeling the ROBETTA integrated method (Supplementary Figure 5).

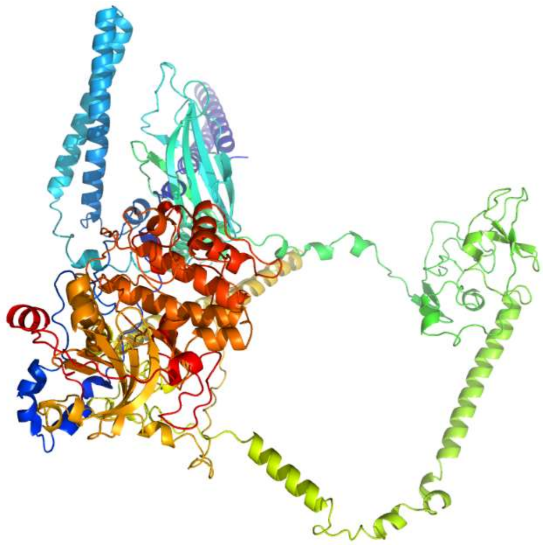

Figure S5. VdPKC protein model constructed by ROBETTA
