# Supplementary material for: Protein Kinase C Is Involved in Vegetative Development, Stress Response and Pathogenicity in Verticillium dahliae
Source: Int J Mol Sci. 2023 Sep 19;24(18):14266. doi: 10.3390/ijms241814266 (PMC10531995; doi:10.3390/ijms241814266)
Supplement: Supplementary file 1 [file ijms-24-14266-s001.zip › Supplementary Figure S6.pdf]

International Journal of
Molecular Sciences

MDPI

Article

Protein Kinase C Is Involved in Vegetative Development, Stress Response and Pathogenicity in *Verticillium dahliae*

Dahui Wang, Zhibo Zhao, Youhua Long and Rong Fan *

College of Agriculture, Guizhou University, Guiyang 550025, China; gzwdh07@126.com (D.W.); zbzhao@gzu.edu.cn (Z.Z.); yhlong3@gzu.edu.cn (Y.L.)
* Correspondence: rfan@gzu.edu.cn

Supplementary Figure S6. Two-dimensional binding patterns of VdPKC protein with dimethomorph, fluormorph and polyoxin

An autodock-vina docking score value of less than -7.0 indicates that the target has strong binding ability with the compound, a score of less than -5.0 greater than -7.0 indicates that the compound small molecule has better binding ability with the target, and a score of between -5.0 and -4.25 indicates that the compound has some binding ability with the macromolecular target. Docking results showed that VdPKC protein could bind well to polyoxin small molecules to form a pocket complex (Supplementary Figure 6). The free binding energies is -5.6 kcJ/mol (Supplementary Table 2). It shows that VdPKC protein has good binding ability with fungicide molecule polyoxin.

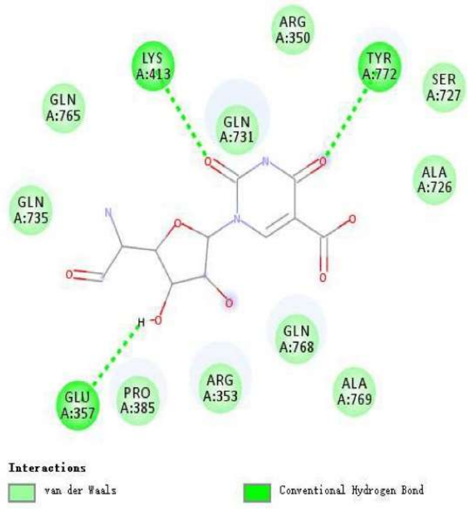

Figure S6. Two-dimensional binding patterns of VdPKC protein with dimethomorph, fluormorph and polyoxin
