# Supplementary material for: Protein Kinase C Is Involved in Vegetative Development, Stress Response and Pathogenicity in Verticillium dahliae
Source: Int J Mol Sci. 2023 Sep 19;24(18):14266. doi: 10.3390/ijms241814266 (PMC10531995; doi:10.3390/ijms241814266)
Supplement: Supplementary file 1 [file ijms-24-14266-s001.zip › Supplementary Table S1.pdf]

International Journal of *Molecular Sciences*

MDPI

Article

Protein Kinase C Is Involved in Vegetative Development, Stress Response and Pathogenicity in *Verticillium dahliae*

Dahui Wang, Zhibo Zhao, Youhua Long and Rong Fan *

College of Agriculture, Guizhou University, Guiyang 550025, China; gzwdh07@126.com (D.W.); zbzhao@gzu.edu.cn (Z.Z.); yhlong3@gzu.edu.cn (Y.L.)

* Correspondence: rfan@gzu.edu.cn

Table S1. Sequences and purpose of the primers used in this study

Primer name	Primer sequence (5'-3')	Purpose
VdPKC-F	GATGCGAAGGAAGAGGATT	Amplification of *VdPKC* gene
VdPKC-R	ATTGCTTGCGACAGAACT	
VdPKC-q-F	AGGAGATGCGAAGGAAGA	Detection of *VdPKC* expression level
VdPKC-q-R	CCAAGAGACAGGCGAATT	
β-tubulin-F	TTTCCAGATCACCCACTCC	Detection of expression of reference gene
β-tubulin-R	ACGACCGAGAAGGTAGCC	
VdPKC-up-F	ccaagctcaagctaagcttCTCTGACGCCATCACAGAGT	Amplification of upstream homologous fragment of *VdPKC* gene
VdPKC-up-R	atgccgaccgggaaccagttAGCTGTCGAGACAGGTCGAT	
VdPKC-down-F	aatatcagttgggctgcaggGAGCCAGGACATGATAGATGACT	Amplification of downstream homologous fragment of *VdPKC* gene
VdPKC-down-R	attaacgccgaattgaattcTCCGTACCTTGTATAAAATGTTGTAAG	
Hyg-F	AACTGGTTCCCGGTCGGCATCTACTC	Amplification of *Hyg* gene
Hyg-R	CCTGCAGCCCAACTGATATTGAAGGAGC	
up-*Hyg*-F	CTCTGACGCCATCACAGAGT	Knockout transformer validation
up-*Hyg*-R	AAATTTTGTGCTCACCGCCTGGAC	
Hyg-down-F	TCTCCTTGCATGCACCATTCCTTG	
Hyg-down-R	TCCGTACCTTGTATAAAATGTTGTAAG	
VdPKC-tg-F	GATGCGAAGGAAGAGGATT	Detection of *VdPKC* target gene fragments
VdPKC-tg-R	ATTGCTTGCGACAGAACT	
Hyg-tg-F	CCTGAACTCACCGCGACGTC	Detection of *Hyg* target gene fragments
Hyg-tg-R	CTATTCCTTTGCCCTCGGACGAGTG	
VdPKC-hb-F	cggccagtgccaagcttCCGGATTAATGCCAACTGCG	Amplification of *VdPKC* and upstream homologous segments
VdPKC-hb-R	gcagcttctgcgaattcTTCAAAGTCGGCCGTGTAG	
VdPKC-cx-F	ACGTCAGAAGCCGACTGC	Amplification of *VdPKC* target gene segments by complementing mutants
VdPKC-cx-R	TGAACTTGTGGCCGTTTACG	
actin-F	GCTTCCCGATGGTCAAGTCA	Detection of expression of reference gene
actin-R	GGATTCCAGCTGCTTCCATTC	
Vd-F	CCGCCGGTCCATCAGTCTCTCTGTTTATAC	

Vd-R	CGCCTGCGGGACTCCGATGCGAGCTGTAAC	Detection of *Verticillium dahliae* biomass
q-VdPks1-F	CTCGTCCTGATCCGTATCCCA	Detection of *VDAG_00190* expression level
q-VdPks1-R	TGTGAATTGAGGCAGGCATG	
q-VdBrn1-F	TGGCATCAAGACAGACATGTA	Detection of *VDAG_03665* expression level
q-VdBrn1-R	ACAACGCGAGCGATGTCGAT	
q-VdScd1-F	AAAGGTGTTTGAGAGCGGAC	Detection of *VDAG_0339*3 expression level
q-VdScd1-R	ATCTCCCTCTCCACAACAGC	
q-VdBrn2-F	TATGTCCCTGGCGGCTTTAA	Detection of *VDAG_00183* expression level
q-VdBrn2-R	TGATCCACTCGCAGTCTTCA	
q-VdCmr1-F	AGTTCAGAGGAAGCCGTCTT	Detection of *VDAG_00195* expression level
q-VdCmr1-R	CAGCGTCGTGACAGTATTCG	
VDH1-F	GTCTATTCATCTGGTTCCTCCCTA	Detection of *VDH1* expression level
VDH1-R	CAAACCTCTTACAATGTTGACGC	

Note: lowercase letters are primer sequences of splices
