# Supplementary material for: Protein Kinase C Is Involved in Vegetative Development, Stress Response and Pathogenicity in Verticillium dahliae
Source: Int J Mol Sci. 2023 Sep 19;24(18):14266. doi: 10.3390/ijms241814266 (PMC10531995; doi:10.3390/ijms241814266)
Supplement: Supplementary file 1 [file ijms-24-14266-s001.zip › Supplementary Table S2.pdf]

International Journal of
Molecular Sciences

MDPI

Article

Protein Kinase C Is Involved in Vegetative Development, Stress Response and Pathogenicity in *Verticillium dahliae*

Dahui Wang, Zhibo Zhao, Youhua Long and Rong Fan *

College of Agriculture, Guizhou University, Guiyang 550025, China; gzwdh07@126.com (D.W.); zbzhao@gzu.edu.cn (Z.Z.); yhlong3@gzu.edu.cn (Y.L.)
* Correspondence: rfan@gzu.edu.cn

Table S2. Molecular docking results of VdPKC protein with fungicide molecule

Fungicides	binding energy (kcal/mol)	Critical amino aaid
Polyoxin	-5.6	TYR772、GLN735、GLN731、GLU357、ARG353、LYS413
